# Estimated Current and Future Congenital Rubella Syndrome Incidence with and Without Rubella Vaccine Introduction — 19 Countries, 2019–2055

**DOI:** 10.15585/mmwr.mm7418a3

**Published:** 2025-05-22

**Authors:** Emmaculate Lebo, Emilia Vynnycky, James P. Alexander, Matthew J. Ferrari, Amy K. Winter, Kurt Frey, Timoleon Papadopoulos, Gavin B. Grant, Patrick O’Connor, Susan E. Reef, Natasha S. Crowcroft, Laura A. Zimmerman

**Affiliations:** ^1^Global Immunization Division, Global Health Center, CDC; ^2^Statistics, Modelling and Economics Department, United Kingdom Health Security Agency, London, United Kingdom; ^3^Department of Infectious Disease Epidemiology and Dynamics, London School of Hygiene & Tropical Medicine, London, United Kingdom; ^4^Center for Infectious Disease Dynamics, The Pennsylvania State University, University Park, Pennsylvania; ^5^Department of Epidemiology & Biostatistics, College of Public Health, University of Georgia, Athens, Georgia; ^6^Institute for Disease Modeling, Gates Foundation, Seattle, Washington; ^7^Independent consultant, Atlanta, Georgia; ^8^Center of Vaccine Preventable Diseases, Dalla Lana School of Public Health, University of Toronto, Toronto, Canada.

**Summary**

**What is already known about this topic?**

Rubella infection during early pregnancy can result in miscarriage, fetal death, or a constellation of birth defects known as congenital rubella syndrome (CRS). By 2023, among 194 World Health Organization member countries, 175 (90%) had introduced a rubella-containing vaccine (RCV) into their routine immunization program.

**What is added by this report?**

In this modeling study of vaccination, in the 19 countries that have not introduced RCV and where an estimated 24,000 CRS cases occurred in 2019, universal RCV introduction during 2025–2055 would avert an estimated 986,000 CRS cases. In 2024, based on these estimates and other considerations, the World Health Organization recommended universal RCV introduction in these 19 countries.

**What are the implications for public health practice?**

Universal RCV introduction during the next 5 years could accelerate progress toward rubella and CRS elimination worldwide.

## Abstract

Rubella is a leading cause of vaccine-preventable birth defects. Rubella virus infection during early pregnancy can result in miscarriage, fetal death, stillbirth, or a constellation of birth defects known as congenital rubella syndrome (CRS). This report describes current and future estimated CRS incidence in countries that have not yet introduced rubella-containing vaccine (RCV) into their national childhood immunization schedules and the estimated effect of implementing a recent recommendation to introduce RCV into these programs even if population coverage with measles-containing vaccine is <80%. During 2000–2022, the number of countries that introduced RCV increased from 99 (52%) of 191 in 2000 to 175 (90%) of 194 in 2022. By the end of 2023, 19 lower- and middle-income countries had not yet introduced RCV. In 2019, an estimated 24,000 CRS cases occurred in these countries, representing 75% of the estimated 32,000 cases worldwide. In a modeling study estimating the effect of RCV introduction in these countries during 2025–2055, an estimated 1.03 million CRS cases are projected to occur without RCV. In contrast, fewer than 60,000 cases are estimated if RCV is introduced with catch-up and follow-up supplementary immunization activities, averting more than an estimated 986,000 CRS cases over 30 years. Based in part on these estimates, in September 2024, the World Health Organization Strategic Advisory Group of Experts on Immunization recommended removing the ≥80% coverage threshold and instituting universal RCV introduction in these countries. RCV introduction in these 19 countries during 2025–2030 could rapidly accelerate progress toward rubella and CRS elimination worldwide.

## Introduction

Rubella is a leading cause of vaccine-preventable birth defects. Rubella virus infection usually produces a mild febrile rash illness in children and adults. However, infection during pregnancy, especially in the first trimester, can result in miscarriage, fetal death, stillbirth, or a constellation of birth defects known as congenital rubella syndrome (CRS). Caring for CRS cases is costly, and rubella vaccination has been shown to be cost-effective in high- and middle-income countries. However, no similar studies have been conducted in low-income countries in Africa or Asia (*1*). A single dose of rubella-containing vaccine (RCV) can provide lifelong protection against rubella (*1*). The World Health Organization (WHO) Global Vaccine Action Plan 2011–2020* included a target to achieve rubella elimination in at least five of the six WHO regions by 2020, and the WHO Immunization Agenda 2030^†^ includes rubella elimination as a critical impact goal.

During 2000–2022, the number of countries that included RCV in their childhood immunization schedules increased from 99 (52%) of 191 in 2000 to 175 (90%) of 194 in 2022 (*2*,*3*). However, the 2020 WHO Rubella Vaccine Position Paper (*1*) maintained WHO’s 2011 recommendation that countries planning to introduce RCV into their immunization programs should have attained ≥80% coverage with the first dose of measles-containing vaccine (MCV1)^§^ through routine vaccination or ≥80% coverage with an MCV dose through supplementary immunization activities (SIAs) (*1*). The rationale for the 80% coverage threshold is to avoid suboptimal RCV postintroduction vaccination coverage, which would have the effect of reducing, but not eliminating, rubella transmission; shifting the age of infection to older children, adolescents, and young adults who were not immune; increasing the interval between rubella outbreaks; and increasing the risk for rubella infection among nonimmune women of childbearing age, potentially leading to a paradoxical increase in cases of CRS compared with the prevaccine rubella epidemiology. Any change to the 80% coverage requirement would require recommendation by the WHO Strategic Advisory Group of Experts (SAGE) on Immunization, the principal independent advisory group to WHO for vaccines.^¶^

The recommended strategy for introducing RCV into national immunization programs is through an initial catch-up SIA using combined measles and rubella (MR) vaccine for all children aged 9 months–14 years, followed immediately by introduction of MR vaccine into the routine childhood immunization schedule (*1*). WHO recommends that countries then achieve and maintain ≥80% coverage with ≥1 dose of RCV (as a combined MR vaccine) delivered through routine services or SIAs (*1*). Follow-up MR SIAs, usually focusing on children aged <5 years, are conducted every 3–4 years to reduce measles susceptibility in the population and prevent outbreaks. This report describes the estimated current and future estimated CRS incidence in the 19 countries** that had not introduced RCV by the end of 2023, and the impact of implementing the September 2024 recommendation by WHO SAGE for universal introduction of RCV in these countries (*4*).

## Methods

### Immunization Activities

Each year, countries report vaccination data to WHO and UNICEF using the electronic Joint Reporting Form (eJRF).^††^ WHO and UNICEF estimate coverage with the first and second MCV doses administered through routine immunization services^§§^ for all countries, using annual administrative coverage data (the number of vaccine doses administered divided by the estimated target population), national coverage estimates, and vaccination coverage surveys. For this report, 2019–2023 eJRF data were reviewed. World Bank income groupings for fiscal year 2024 (based on July 2023 data)^¶¶^ and the World Bank index of fragile and conflict-affected countries for 2023*** were used to categorize countries’ income and vulnerability status.

### Rubella and CRS Surveillance

Rubella surveillance relies on the measles surveillance system to detect cases of febrile rash illness. Rubella cases reported through eJRF using a standard case definition were reviewed for this report (*3*). CRS cases, which are also reported through eJRF using a standard case definition, are detected through separate surveillance systems, often using sentinel sites that might not be nationally representative (*3*).

### Modeled Estimates of Current CRS Incidence

Because routine surveillance data underestimate CRS cases, estimates of CRS incidence (cases per 100,000 live births) and the number of annual CRS cases in 2019 for the 19 countries that had not yet introduced RCV by the end of 2023 (calculated as part of published estimates of the global and regional burden of CRS during 1996–2019) were used (*5*). These published estimates were calculated using 1) previously described catalytic models to estimate the age-specific prevaccination force of infection (the rate at which susceptible persons become infected) from seroprevalence data and 2) an age-structured dynamic rubella transmission model (*5*). For each dataset and catalytic model, 95% CIs for the force of infection, and when applicable, assay sensitivity, were derived. For each country, the transmission model was run using 1,000 values for the prevaccination force of infection, vaccine efficacy, vaccination coverage, and risk that an infant would be born with CRS if the mother was infected with rubella during pregnancy. These parameters were varied in the same range as that used previously (*6*). The base case value for vaccine efficacy was 95%, with a range of 85%–99%, and the assumed risk that a child born to a mother who was infected during pregnancy would have CRS was 65% (95% CI = 47%–88%), consistent with published estimates (*5*). The model used demographic data from United Nations population sources.^†††^ The 95% CIs for each outcome of interest were calculated from the outcome’s range across the 1,000 model runs.

### Modeled Estimates of Future CRS Incidence

To estimate the impact of RCV introduction on future CRS cases, the dynamic rubella transmission model (*5*) was adjusted to account for rubella virus importations and correlations between vaccine doses, and run for each of the 19 countries under three scenarios: 1) no RCV introduction in any country; 2) RCV introduction in 2025, beginning with a wide age-range catch-up MR SIA (for children aged 9 months–14 years) and continuing with follow-up MR SIAs (for children aged 9 months–4 years) every 4 years, with 90% MR vaccination coverage; and 3) RCV introduction in 2025, with a wide age-range catch-up and follow-up SIAs at the same intervals as the previous scenario, but with 60% (rather than 90%) MR vaccination coverage. For all three scenarios, the model was run 200 times (consistent with previous work for the Vaccine Impact Modeling Consortium and work on measles and rubella elimination), using the mean of 2018 and 2019 prepandemic MCV1 coverage as a proxy for future routine MR vaccination coverage and held constant over time.

To estimate the number of CRS cases averted by RCV introduction, the number of CRS cases under each of the vaccination scenarios was subtracted from the number in the no-vaccination scenario. As a sensitivity analysis, all scenarios were run using an independently developed University of Georgia (UGA) model. Although the two models varied in some of their assumptions,^§§§^ the two models generated similar outputs about the 30-year percentage reduction in CRS cases. Therefore, modeling findings are included from only the United Kingdom Health Security Agency (UKHSA) dynamic rubella transmission model for simplicity. This activity was reviewed by CDC, deemed not research, and was conducted consistent with applicable federal law and CDC policy.^¶¶¶^

## Results

### Immunization Activities and Eligibility to Introduce RCV

**FIGURE 1:**
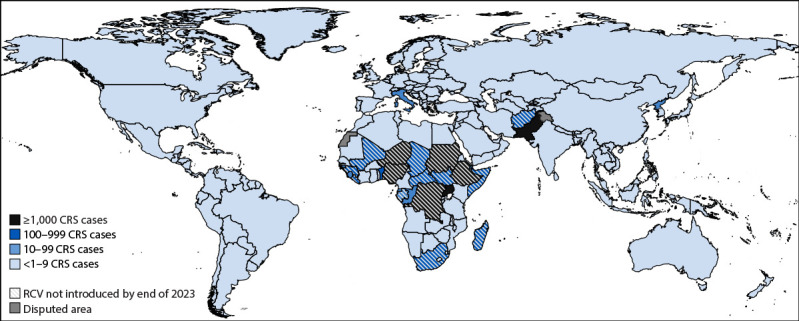
Estimated number of congenital rubella syndrome cases, 2019* and introduction of rubella-containing vaccine, by country, 2023 — worldwide^†^^,^^§^^,^^¶^ **Abbreviations:** CRS = congenital rubella syndrome; RCV = rubella-containing vaccine; WHO = World Health Organization. * Based on Estimates of the global burden of congenital rubella syndrome, 1996-2019. ^†^ Introduction of RCV into the routine immunization program by December 31, 2023, as reported to WHO and UNICEF using the electronic Joint Reporting Form. ^§^ By the end of 2023, 19 countries had not introduced RCV into their routine immunization program: Afghanistan, Central African Republic, Chad, Democratic Republic of the Congo, Djibouti, Equatorial Guinea, Ethiopia, Gabon, Guinea, Guinea-Bissau, Liberia, Madagascar, Mali, Niger, Nigeria, Somalia, South Africa, South Sudan, and Sudan. In 2019, when the estimates of CRS cases were made, Comoros, Pakistan, and Uganda had also not introduced RCV but did so by the end of 2023. ^¶^ Italy introduced RCV in 1972, initially only for girls aged 11–12 years, and RCV was introduced into routine immunization in 1990; because of historic coverage levels, CRS still poses a potential risk.

By the end of 2023, RCV had been introduced into the childhood immunization programs of all but 19 (10%) of 194 countries (Figure 1). Among these 19 countries, 15 are in the WHO African Region, and four are in the WHO Eastern Mediterranean Region; 13 are classified as low income and 12 are classified as either conflict-affected or having fragile institutions (Table).

**TABLE:** Estimated measles-containing vaccine coverage, reported rubella incidence, and estimated congenital rubella syndrome incidence among countries that had not introduced rubella-containing vaccine by December 2023, by country and World Health Organization region — 19 countries, 2019 and 2023

Region/Country	World Bank income group,* 2023	FCV setting,^†^ 2023	MCV1 coverage, %^§^		Most recent measles SIA^§^		Met 80% MCV coverage required for RCV introduction,^¶^ 2023	No. of reported rubella cases^§^		Rubella incidence**		Estimated no. of CRS cases^†† ^(95% CI), 2019	Estimated CRS incidence^§§^ (95% CI), 2019
			2019	2023	Year	MCV SIA coverage, %^¶¶^		2019	2023	2019	2023		
**African Region**													
Central African Republic	LIC	Conflict	41	41	2023	NR	No	182	121	368	235	172 (<1–603)	105 (<1–367)
Chad	LIC	Fragile	40	63	2023–24	NR	No	24	10,918	14	5,651	688 (<1–2,347)	105 (<1–357)
Democratic Republic of the Congo	LIC	Conflict	65	52	2023–24	95	Yes	561	650	60	61	2,771 (<1–8,828)	80 (<1–253)
Equatorial Guinea	UMIC	NA	51	61	2020	ND	No	3	3	18	16	44 (<1–159)	101 (<1–363)
Ethiopia	LIC	Conflict	57	61	2023	88	Yes	266	1,085	23	84	3,507 (<1–10,125)	99 (<1–283)
Gabon	UMIC	NA	62	66	2017	ND	No	0	7	0	28	81 (44–125)	121 (66–188)
Guinea	LMIC	NA	47	47	2022	ND	No	80	67	61	47	465 (<1–1,605)	102 (<1–352)
Guinea-Bissau	LIC	Fragile	79	72	2019	88	Yes	0	255	0	1,184	63 (<1–217)	96 (<1–330)
Liberia	LIC	NA	68	82	2018	89	Yes	140	NR	278	NR	164 (<1–577)	102 (<1–360)
Madagascar	LIC	NA	60	51	2022	65	No	122	390	43	125	697 (<1–1,945)	80 (<1–223)
Mali	LIC	Conflict	71	73	2019	84	Yes	19	15	9	6	824 (<1–2,856)	103 (<1–358)
Niger	LIC	Conflict	79	80	2022	92	Yes	7	88	3	34	1,102 (<1–3,786)	105 (<1–360)
Nigeria	LMIC	Conflict	58	60	2022–23	87	Yes	1,644	10,221	78	449	9,719 (<10–25,102)	130 (<1–337)
South Africa	UMIC	NA	83	80	2023	ND	Yes	1,370	870	230	138	422 (22–1,095)	36 (1.9–93)
South Sudan	LIC	Conflict	65	72	2023	ND	No	149	44	143	38	504 (<1–1,365)	79 (<1–214)
**Eastern Mediterranean Region**													
Afghanistan	LIC	Conflict	57	55	2022	NR	No	59	444	16	107	914 (<1–2,477)	77 (<1–207)
Djibouti	LMIC	NA	83	76	2020	ND	No	NR	5	NR	43	12 (<1–32)	59 (<1–157)
Somalia	LIC	Conflict	46	46	2022	NR	No	0	2,407	0	1,311	358 (<1–1,222)	94 (<1–321)
Sudan	LIC	Fragile	90	51	2019	98	Yes	281	399	62	80	1,230 (<1–2,727)	91 (<1–202)

**Abbreviations:** CRS = congenital rubella syndrome; FCV = fragile, conflict-affected, or vulnerable; LIC = low-income country; LMIC = lower-middle–income country; MCV1 = first dose of measles-containing vaccine; MCV2 = second dose of measles-containing vaccine; NA = not applicable; ND = not done; NR = not reported; RCV = rubella-containing vaccine; SIA = supplementary immunization activity; UMIC = upper-middle–income country; WHO = World Health Organization.

* Gross national income per capita in U.S. dollars for fiscal year 2024: high income ≥$13,846; UMIC = $4,466–$13,845; LMIC = $1,136–$4,465; and LIC ≤$1,135. World Bank Country and Lending Groups – World Bank Data Help Desk

^†^ Developed by the World Bank, the Classification of Fragile and Conflict-Affected Situations is a framework to categorize countries based on their risk for conflict, fragility, and weak governance. Two categories are used: 1) countries with high levels of institutional and social fragility, identified on the basis of indicators that measure the quality of policy and institutions, and manifestations of fragility; and 2) countries affected by violent conflict, identified on the basis of a threshold number of conflict-related deaths relative to the population. Classification of Fragile and Conflict-Affected Situations

^§^ Measles vaccination coverage

^¶^ In 2020, WHO confirmed its recommendation that countries achieve a coverage of ≥80% with MCV1 through routine vaccination or with a dose of MCV during SIAs before introducing RCV into the routine immunization schedule. Rubella vaccines: WHO position paper - July 2020

** Rubella incidence is calculated on the basis of cases reported through the WHO and UNICEF electronic Joint Reporting Form and United Nations population data. Number of cases are calculated per 1 million population.

^††^ Based on a dynamic transmission model, developed at the United Kingdom Health Security Agency. Estimates of the global burden of Congenital Rubella Syndrome, 1996-2019 - ScienceDirect

^§§^ Cases per 100,000 live births.

**^¶¶^** MCV doses administered through SIAs are considered supplementary doses and are not counted toward MCV1 or MCV2 coverage. Coverage results are based on validated postcampaign coverage surveys, using the WHO coverage survey methodology. World Health Organization Vaccination Coverage Cluster Surveys: Reference Manual

In 2019, before the COVID-19 pandemic, estimated routine MCV1 coverage in all 19 countries ranged from 40% to 90%. During the pandemic period (2020–2022), 12 of these countries reported declines in MCV1 coverage, ranging from 2% to 33%. By 2023, MCV1 coverage had recovered to prepandemic levels in most countries (range = 41%–82%); however, only three (Liberia, Niger, and South Africa) achieved ≥80% MCV1 coverage through routine vaccination services. During 2017–2023, 18 countries conducted at least one nationwide SIA, eight (44%) of which achieved MCV coverage of ≥80% based on a validated postcampaign coverage survey. By the end of 2023, nine (47%) of the 19 countries reached the 80% MCV coverage threshold, either through routine vaccinations or SIAs, and thereby became eligible for RCV introduction.

### Surveillance Activities and Reported Rubella Incidence

In 2023, these 19 countries reported 27,989 (78%) of the 35,714 reported rubella cases worldwide (Table). The countries with the highest annual reported incidences were Chad (5,651 cases per 1 million population), Guinea-Bissau (1,184), and Somalia (1,311).

### Modeled Estimates of Current and Future CRS Cases

In 2019, an estimated 24,000 CRS cases occurred in the 19 countries that had not yet introduced RCV by 2023, accounting for 75% of an estimated 32,000 CRS cases worldwide. Approximately 1,000 CRS cases were estimated to have occurred in each of five countries (Democratic Republic of the Congo, Ethiopia, Niger, Nigeria, and Sudan) (Figure 1).

**FIGURE 2:**
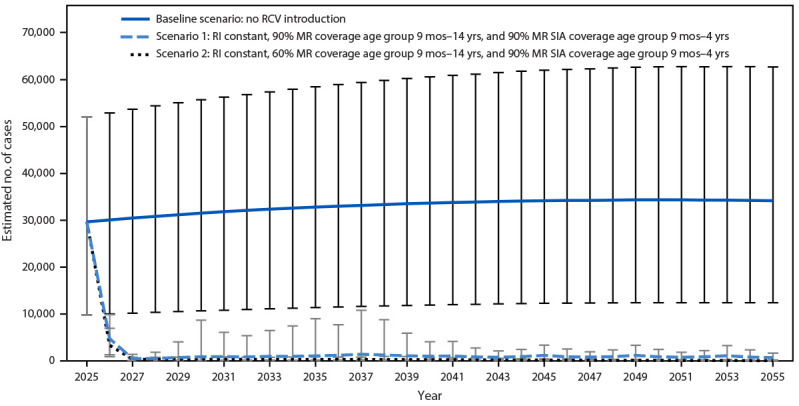
Estimated number of annual congenital rubella syndrome cases with rubella vaccine introduction and in the absence of rubella vaccine introduction — 19 countries, 2025–2055*^,^^†^^,^^§^ **Abbreviations:** CRS = congenital rubella syndrome; MCV1 = first dose of measles-containing vaccine; MR = measles and rubella; RCV = rubella-containing vaccine; RI = routine immunization; SIA = supplementary immunization activity; WHO = World Health Organization. * With 95% CIs indicated by bars. ^†^ In the two vaccination scenarios, rubella vaccine is introduced by combining with measles-containing vaccine, and annual RI coverage with MR vaccine remains at the mean of the 2018 and 2019 estimates for MCV1 for 2025–2055. In each scenario, MR introduction is preceded by a wide-age catch-up MR SIA for children aged 9 months–14 years as recommended by WHO, and then followed every 4 years by a follow-up MR SIA for children aged 9 months–4 years, which is needed for measles elimination. In the first scenario, coverage with both types of SIAs is 90%; in the second, 60%. ^§^ CRS cases prevented by rubella vaccine introduction and vaccination are estimated by the area between the estimated cases with vaccination and those without vaccination. A cumulative total of 986,000 cases were estimated to have been prevented by rubella vaccination in either of the two scenarios during 2025–2055 in the 19 countries: Afghanistan, Central African Republic, Chad, Democratic Republic of the Congo, Djibouti, Equatorial Guinea, Ethiopia, Gabon, Guinea, Guinea-Bissau, Liberia, Madagascar, Mali, Niger, Nigeria, Somalia, South Africa, South Sudan, and Sudan.

Modeling estimates indicated that in the absence of RCV introduction, a mean of 30,000–34,000 CRS cases would occur each year during 2025–2055, with a cumulative mean of 1,025,286 cases across all 19 countries during this 30-year period (Figure 2). In contrast, with RCV introduction in the 19 countries in 2025, the estimated annual number of CRS cases would decline sharply during 2025–2027 in both vaccination scenarios (i.e., 60% and 90% MR coverage) and remain low through 2055. By 2055, the projected cumulative mean number of cases in both vaccination scenarios is estimated to be 40,000–60,000. During 2025–2055, compared with no RCV introduction, rubella vaccination would prevent an estimated 986,000 CRS cases in these 19 countries.

## Discussion

The 19 countries that have not yet introduced RCV are home to some of the world’s most vulnerable populations and communities and include approximately 25 million infants (*3*). These countries account for most of the current global rubella and CRS cases, with CRS incidences comparable to those found worldwide in the prevaccine era (*5*). RCV introduction in these countries could avert an estimated 30,000 CRS cases annually and approximately 986,000 cases during the next 30 years.

Other recent modeling studies have documented that RCV introduction into routine immunization programs in these and other countries has the immediate effect of decreasing rubella and CRS cases, resulting in a lower CRS incidence than would occur without vaccination for approximately 15 years even without introductory SIAs, and can achieve elimination of rubella transmission with improved routine coverage or moderate coverage SIAs (*7*–*9*). The projected rapid and sustained reductions in CRS cases that would follow RCV introduction in these 19 countries supports universal RCV introduction.

To determine whether ≥80% MCV coverage should continue to be required before a country is eligible to introduce RCV, the findings from the two modeling groups underwent a methodologic review by the WHO Immunization and Vaccines-Related Research Advisory Committee in June 2024 (*10*). At the September 2024 SAGE meeting, the evidence on rubella epidemiology, routine and SIA coverage with MCV in countries currently not using RCV, the programmatic success of RCV introduction in other countries, and estimated future incidence from mathematical models were presented by the Measles and Rubella Partnership**** Rubella Task Team. Responding to these findings, SAGE recommended removing the 80% coverage threshold, called for universal introduction of RCV in the remaining 19 countries, and continued the recommendation to conduct wide age-range catch-up SIAs with MR vaccine before introduction to ensure that those persons who missed earlier vaccination opportunities are protected (*4*). The SAGE recommendations have set the stage to facilitate progress toward the Global Vaccine Action Plan 2011–2020 and Immunization Agenda 2030 rubella elimination goals.

To capitalize on this new opportunity, the remaining 19 countries will need support from global partners to facilitate vaccine introduction, with a particular focus on ensuring high-quality SIAs. Support from Gavi, the Vaccine Alliance,^††††^ for low- and lower-middle–income countries is crucial to ensure access to vaccines and to facilitate building the infrastructure necessary for effective delivery during these SIAs. Also critical is that countries not eligible for Gavi support receive support to introduce RCV, ensuring that global elimination can be achieved. In fragile and conflict-affected settings, robust monitoring and implementation strategies will be needed to ensure that SIAs reach all populations and reduce the incidence of rubella and CRS.

### Limitations

The findings in this report are subject to at least three limitations. First, the quality of data on measles vaccination coverage, including SIA coverage, as well as on reported rubella cases, can vary and might lead to inaccurate estimates, making accuracy and reliability assessments challenging. Second, although the prospective modeling assumed that all 19 countries would introduce RCV in 2025, the year of introduction will likely vary during the next ≥5 years for each country; thus, the impact over time will vary from these estimates. Finally, the modeled estimates depend on data inputs that can vary in quality and completeness, as well as the model assumptions, potentially influencing the accuracy of the projections.

### Implications for Public Health Practice

The decision by SAGE to recommend universal RCV introduction in the remaining countries provides an opportunity to reduce the global incidence of CRS, a preventable and potentially fatal condition, and to eliminate barriers to vaccine introduction resulting from the current 80% MCV coverage threshold. RCV introduction in these 19 countries during 2025–2030 will rapidly accelerate progress toward rubella and CRS elimination worldwide.

## Footnotes

*
Global vaccine action plan 2011-2020

†
Immunization Agenda 2030

§ Achieving ≥80% coverage with MCV1 during routine vaccinations or a supplementary dose of MCV during SIAs is used as a means to demonstrate the country's ability to achieve these levels of RCV coverage following introduction, because RCV is administered as a combined measles-rubella or measles-mumps-rubella vaccine.

¶
Strategic Advisory Group of Experts on Immunization

** Afghanistan, Central African Republic, Chad, Democratic Republic of the Congo, Djibouti, Equatorial Guinea, Ethiopia, Gabon, Guinea, Guinea-Bissau, Liberia, Madagascar, Mali, Niger, Nigeria, Somalia, South Africa, South Sudan, and Sudan.

††
Immunization, Vaccines and Biologicals

§§
WHO Immunization Data portal - Global

¶¶ The World Bank publishes annual gross national income classification cutoffs per capita in U.S. dollars. The 2024 fiscal year provides classification data through 2023: high income ≥$13,846; upper-middle income = $4,466–$13,845; lower-middle income = $1,136–$4,465; and low income ≤$1,135. World Bank Country and Lending Groups – World Bank Data Help Desk

*** Developed by the World Bank, the Classification of Fragile and Conflict-Affected Situations is a framework to categorize countries based on their vulnerability to conflict, fragility, and weak governance. Two categories are used: 1) countries with high levels of institutional and social fragility, identified based on indicators that measure the quality of policy and institutions, and manifestations of fragility; and 2) countries affected by violent conflict, identified based on a threshold number of conflict-related deaths relative to the population. Classification of Fragile and Conflict-Affected Situations

†††
United Nations Data Portal Population Division

§§§ To assess sensitivity model structure, all RCV introduction scenarios were run using a second model developed at UGA. The UGA model 1) allows for nonstable populations with changing birth and death rates, whereas the UKHSA model assumes stable population with fixed birth rates and then scales outputs to population projections; 2) incorporates transmission seasonality, whereas the UKHSA model does not; and 3) is not sex-structured, whereas the UKHSA model is sex-structured (i.e., considers population dynamics of males and females separately, in terms of their distinct biologic processes).

¶¶¶ 45 C.F.R. part 46.102(l)(2), 21 C.F.R. part 56; 42 U.S.C. Sect. 241(d); 5 U.S.C. Sect. 552a; 44 U.S.C. Sect. 3501 et seq.

****
Measles & Rubella Partnership

†††† Gavi is an international organization bringing together public and private sectors with the shared goal of saving lives and protecting health by increasing equitable and sustainable use of vaccines. Eligibility for Gavi's financial support is based on national income. In 2025, countries became eligible for Gavi support if their most recent gross national income per capita, as reported by the World Bank, was ≤$1,820 U.S. dollars. Since 2011, the eligibility threshold is adjusted for inflation on an annual basis.
